# Comparison of Antioxidant and Antibacterial Activities of Camellia Oil From Hainan With Camellia Oil From Guangxi, Olive Oil, and Peanut Oil

**DOI:** 10.3389/fnut.2021.667744

**Published:** 2021-05-03

**Authors:** Lanying Wang, Shakil Ahmad, Xi Wang, Hua Li, Yanping Luo

**Affiliations:** College of Plant Protection, Hainan University, Haikou, China

**Keywords:** camellia oil from Hainan, camellia oil from Guangxi, olive oil, peanut oil, *Saccharomyces cerevisiae*

## Abstract

**Background/Aim:** Camellia oil from Hainan (SY) is a unique vegetable oil in Hainan, China, due to the geographical environment and oil extraction only through simple physical treatments. To compare SY with camellia oil from Guangxi (SC), olive oil (GL), and peanut oil (HS), this study analyzed the antioxidant and antibacterial activity of four vegetable oils.

**Methods:** Using Gallic acid, BHT as the control, *Saccharomyces cerevisiae* as the model organism, the antioxidant activities of vegetable oils were measured *in vitro* and *in vivo*, and the antibacterial activity was measured with the minimum inhibitory concentration (MIC) method.

**Results:** The major contents of SY, SC, and HS were oleic Acid; the major content of GL was squalene. The highest total flavonoids content of SY was 39.50 ± 0.41 mg RE/g DW; and the highest total phenolic content of SC was 47.05 ± 0.72 mg GAE/g DW. SY exhibited the strongest scavenging activity of hydroxyl radical (HO·) and superoxide anions (${\text{O}}_{2}^{-}\cdot$), the IC_50_ value were 2.06 mg/mL, 0.62 mg/mL, respectively; and SC showed the strongest DPPH· and ABTS· scavenging activity and the reducing abilities. SY showed excellent effect on survival rate, protection rate, flavonoids uptake of *S. cerevisiae* cells, decreased MDA content and ROS level, inhibited CAT, POD, and GR enzyme activity. The absorption of SC total phenols was the highest by cells. The activity showed GL had a broad-spectrum antibacterial activity.

**Conclusion:** Thus, SY shows potential antioxidant activity and provides an important reference value for people to choose edible vegetable oils.

## Introduction

*Camellia oleifera*, known as the edible tea oil tree, is a subtropical evergreen tree distributed in China and Southeast Asian countries (1, 2). The camellia is the most well-known and largest genus in the Theaceae family, with more than 120 recognized tree species (3). It has been widely grown as an oil crop in many countries including China, the Philippines, India, Brazil, and South Korea (4). More than 90% of the world's production of Camellia oil comes from China (5, 6). The Compendium of Materia Medica recorded that Camellia oil was tasted sweet, lubricating the intestines (7), and clearing away heat and eliminate dampness, moisturizing the lungs and eliminating phlegm. Camellia oil also has good effects on reducing swelling, relieving itching, cuts, burns, and bruises (8). In addition, it was use for the treatment of skin diseases such as dermatitis and stretch marks (9).

Camellia oil is rich in unsaturated fatty acids (2, 10), such as oleic acid, linoleic acid; Sesamin (11), saturated acids (12), polyphenols (13) also are found in camellia oil. These compounds shows excellent antioxidant activities. So, Lee and Yen found methanol extract of camellia oil exhibited DPPH· scavenging activity (11); Wang et al. (10) and Wang et al. (13) reported Supercritical Carbon Dioxide extract of camellia oil also showed a stronger DPPH· and ABTS· scavenging activity; the antioxidant activities from the literatures were most limited in DPPH· and ABTS· scavenging activity *in vitro*, were not involved to treat with *Saccharomyces cerevisiae in vivo*.

The unsaturated and saturated fatty acids of camellia oil were similar to those in olive oil (14), olive oil also showed excellent activities, which could accelerate the elimination of ROS (15, 16), reduce the risk of cardiovascular disease, extend lifespan (17), improve memory and cognitive function in the elderly, and reduce the risk of Alzheimer's (18). And peanut oil is a widely used vegetable oil in China. It is rich in natural vitamin E, unsaturated fats, phytosterols. Its α-tocopherol and γ-tocopherol have antioxidation and anti-aging (19, 20). Consumption of phytosterols may be induced activity of antioxidant enzymes and reduced oxidative stress (21).

Camellia oil from Hainan Island is unique due to the unique geographical environment and oil extraction only through simple physical treatments such as precipitation and filtration. Therefore, the taste and quality of Camellia oil from Hainan are better than ordinary Camellia oil (22). So, we used Camellia oil from Hainan, camellia oil from Guangxi, olive oil from Guangdong and peanut oil from Shandong Province as research samples to compare the *in vitro* antioxidant activities, and evaluate the antioxidant capacity of wild-type *S. cerevisiae*, a good model organism in antioxidant research (23, 24), and genetically deficient *S. cerevisiae in vivo*, also, the antibacterial activity of the four vegetable oils was detected. We hope that these data can provide a reference for people to choose edible vegetable oils.

## Materials and Methods

### Test Materials

#### Vegetable Oils

The tea seeds from Wenchang and Qionghai of Hainan Province were picked and mixed together at December 16, 2018, and dried, shelled, crushed; then roasted, physically pressed at January 7, 2019, precipitated and filtered to yield Camellia oil from Hainan Province (SY); According to above method, camellia oil from Baise of Guangxi Province (SC) were yield at March 5, 2019. A bottle of SY and SC was randomly selected and stored at 25°C. Olive oil (GL) from Guangdong Province (Production date: 2018.10.16) and peanut oil (HS) from Shandong Province (Production date: 2018.06.22) were commercially available and randomly selected in the shelf.

#### Test Strain

The wild-type (WT) BY4741 of *S. cerevisiae* and its homologous gene-deficient strains Sod1 and Ctt1 were provided by the Laboratory of Biotechnology and Food Science, Tianjin University of Commerce (Sod1 encodes cytoplasmic superoxide dismutase, Carries the gene SOD 1 knocked out by the gene KanMX 4; *Ctt*1 carries the gene CAT 1 knocked out by the gene KanMX 4).

#### Test Agent

Folin-Ciocalteu reagent, gallic acid, rutin, 2,6-ditert-butyl-4-methylphenol (BHT), 1,1-diphenyl-2-trinitrophenylhydrazine (DPPH), 2,2-diazo-bis (3-ethyl-benzothiazole-6-sulfonic acid) diammonium salt (ABTS), tripyridyltriazine (TPTZ), isopropyl myristate (IPM), Span80, Tween 80, salicylic acid, trichloroacetic acid, ferric chloride, ferrous sulfate heptahydrate, potassium peroxodisulfate, potassium ferricyanide, etc. were all purchased In Shanghai Maclean Biochemical Technology Co., Ltd. Superoxide dismutase (SOD), peroxidase (POD), catalase (CAT), glutathione reductase (GR) and MDA content kits were purchased from Beijing Soleibao Technology Co., Ltd. All reagents used in this study either analytical or chemical grade were commercially available.

#### Preparation of Vegetable Oils

We prepared four different kinds of vegetable oils using Shah Method (25). Mixing vegetable oils (0.20 g) and 0.40 g IPM as oil phases; 0.58 g Tween 80, 0.14 g Span 80, and 0.18 g *n*-butanol as surfactant; the surfactant was mixed with oil phases in the ratio of 6:4, then were diluted in distilled water to yield the concentration 20 mg/mL.

#### GC-MS Analysis of Vegetable Oils

Take 0.1 g of vegetable oil separately; dilute n-hexane into a 10 mL volumetric flask to dissolve entirely. the vegetable oils components were analyzed by using GC-MS (Agilent 7890B-7000B, USA) technology. Gas chromatographic conditions: Agilent HP-5ms (30 m × 250 μm × 0.25 μm) chromatographic column, temperature program: 60°C (retention 1 min), 6°C/min to 300°C (retention 16 min); carrier gas: He, inlet temperature: 270°C, transfer line temperature: 280°C. Conditions of mass spectrometry: EI source; ionization voltage: 70 eV, ion source temperature: 230°C, scanning range: 20–500 aum, injection volume: 1.0 μL.

#### Determination of Total Phenols Content (TPC)

TPC of vegetable oils was determined according to the method (26). A mixture of 80 μL vegetable oil solution, 200 μL Folin-ciocalteu reagent, and 3,780 μL Na_2_CO_3_ (2% w/v) solution was incubated at 40°C for 60 min, and then measured the absorbance at 760 nm. Gallic acid was used as a positive control. According to the standard curve of gallic Acid, TPC was calculated (mg GAE/g DW, the total phenols equivalent per gram of vegetable oils sample).

#### Determination of Total Flavonoids Content (TFC)

TFC of vegetable oils was determined using the previously described method. We mixed 0.5 mL of vegetable oils, 2.5 mL of ultrapure water and 150 μL of 5%(w/v) sodium nitrite solution, and were kept for 6 min at room temperature, then were added into 300 μL of 10%(w/v) aluminum chloride aqueous solution, keeping at room temperature for 5 min again. We added 1 mL of 1 mol/L sodium hydroxide aqueous solution and 550 μL of ultrapure water, and then the absorbance was measured at 510 nm. Rutin was used as a positive control. According to Rutin's standard curve, TFC was calculated (mg RE/g DW, that is, the equivalent of total flavonoids per gram of vegetable oils).

### Determination of Antioxidant Activity of Vegetable Oils *in vitro*

#### DPPH Radical Scavenging Activity

DPPH scavenging activity of vegetable oils was tested according to the reference (27), with slight modification. For the Preparation of 2 × 10^−4^ M DPPH 95% solution, we used 2 mL of DPPH methanol solution in a test tube, and were mixed with 2 mL of vegetable oils (10, 5, 2.5, 1.25, 0.625 mg/mL). Then were incubated at 25°C in the dark for 30 min. the absorbance A was measured at 517 nm. All experiment was replicated three times. The DPPH· scavenging rate was calculated using the following formula:

DPPH· scavenging rate /% = [1-(Ai-Aj)/A0] × 100

A0 was the absorbance of 2 mL DPPH methanol solution and 2 mL ultrapure water; Ai was the absorbance of 2 mL DPPH methanol solution and 2 mL vegetable oil; Aj was the absorbance of 2 mL ultrapure water and 2 ml vegetable oil.

#### ABTS Radical Scavenging Activity

ABTS· scavenging activity of vegetable oils was measured according to the reference. A mixture of 10 mL 7 mmol/L ABTS radical and 10 mL 2.45 mmol/L potassium persulfate, were stored in the dark for 12–16 h. Two mL vegetable oils (20, 10, 5, 2.5, 1.25 mg/mL) and 2 mL ABTS solution reacted for 6 min, then the absorbance A was measured at 734 nm. The calculation formula of the ABTS scavenging rate was as follows:

ABTS· scavenging rate/% = [1-(Ai-Aj)/A0] × 100

A0 was the absorbance of 2 mL ABTS solution and 2 mL ultrapure water; Ai was the absorbance of 2 mL ABTS solution and 2 mL vegetable oil; Aj was the absorbance of 2 mL ultrapure water and 2 ml vegetable oil.

#### Hydroxyl Radical Scavenging Activity

According to the reference (28), a mixture of 2 mL vegetable oils (20, 10, 5, 2.5, 1.25 mg/mL), 2 mL of 6 mM ferrous sulfate solution, and 2 mL of 6 mM H_2_O_2_ solution were kept at room temperature for 10 min. We added 2 mL of 6 mM salicylic acid, and were kept again for 30 min. The absorbance A of the mixture was measured at 510 nm. Each experiment was replicated three times. HO· scavenging rate was calculated using the formula:

HO· scavenging rate /% = [1-(Ai-Aj)/A0] × 100

Where: A0 was the absorbance of 2 mL ultrapure water, 2 mL ferrous sulfate solution, 2 mL H_2_O_2_ solution, and 2 mL salicylic acid; Ai was the absorbance of 2 mL vegetable oil, 2 mL ferrous sulfate solution, 2 mL H_2_O_2_ solution, and 2 mL salicylic acid; Aj was the absorbance of 2 mL vegetable oil, 2 mL ferrous sulfate solution, 2 mL H_2_O_2_ solution and 2 mL ultrapure water.

#### Superoxide Anion Free Radical Scavenging Activity

According to the reference (29), we mixed 4.5 mL of Tris-HCl buffer solution and 3 mL of ultrapure water. The mixture was incubated at 25°C for 20 min, then added 0.4 mL of 3 mM pyrogallol solution and 1 mL vegetable oils (20, 10, 5, 2.5, 1.25 mg/mL), again incubated at 25°C for 4 min, then immediately 0.1 mL of 8 M hydrochloric acid solution were added to stop the entire reaction. The absorbance A was measured at 325 nm. Each experiment was replicated three times. ${\text{O}}_{2}^{-}\cdot$ scavenging rate was calculated as follows:

${\text{O}}_{2}^{-}\cdot$ scavenging rate /% = [1-(Ai-Aj)/A0] × 100

Where: A0 was the absorbance of 4.5 mL buffer solution, 3 mL ultrapure water, 0.4 mL pyrogallol solution, 1 mL buffer solution, and 0.1 mL hydrochloric acid solution; Ai was the absorbance of 4.5 mL buffer solution, 3 mL ultrapure water, 0.4 mL pyrogallol solution, 1 ml vegetable oil and 0.1 mL hydrochloric acid solution; Aj was the absorbance of 4.5 mL buffer solution, 3 mL ultrapure water, 0.4 mL ultrapure water, 1 mL vegetable oil and 0.1 mL hydrochloric acid solution.

#### FRAP Assay

We performed FRAP assay according to the described method (30), 0.2 mL vegetable oils were mixed with 3.8 mL of FRAP solution (31), incubated at 37°C for 5 min; the absorbance was measured at 593 nm. Ferrous sulfate heptahydrate was used as a positive control. According to the standard curve of ferrous sulfate heptahydrate, the reducing power of vegetable oils was calculated (μM Fe^2+^/g DW, that is, the reducing ability of high-valent iron ions per gram of vegetable oil).

#### Total Reduction Capacity (TRC)

According to the method (32), 1 mL of vegetable oils (20, 10, 5, 2.5, 1.25 mg/mL) was added to 2.5 mL of phosphate buffer with pH = 6.6 and 2.5 mL of 1% (w/v) Potassium ferricyanide solution, and were incubated at 50°C for 20 min, then we added 2.5 mL of 10% (w/v) trichloroacetic acid solution, and were mixed by hand slowly and centrifuged at 3,000 rpm/min for 10 min. After centrifuge 2.5 mL of supernatant was shifted to a new test tube, added 2.5 mL ultrapure water and 0.5 ml 0.1% (w/v) ferric chloride solution, then were incubated for 5 min at 25°C, at last the absorbance was measured at 700 nm.

### Determination of Antioxidant Activity of Vegetable Oils *in vivo*

#### Determination of the Survival Rate of *S. cerevisiae* Cells

Cell survival is expressed as cell tolerance (33). According to the reference, the yeast cells suspension was prepared with YPD (Yeast extract peptone dextrose) medium (34, 35). Ten-milliliter cell suspension was mixed with 40 mL liquid YPD medium thoroughly, adding 200 μL H_2_O as the H_2_O treatment group, 200 μL vegetable oils as the oils group, 200 μL vegetable oil solvent as the solvent group, and no additive as the blank control group. After shaking and mixing thoroughly, the solution was incubated for 1 h at 28°C/180 rpm. Then, 20 μL of H_2_O_2_ (final concentration of 2.0 mM) was added into the above mixtures, set at 28°C/180 rpm for 1 h. The mixtures were diluted 1,500 times, 100 μL of the diluted solution was coated on the plate containing 2% agar YPD medium. The plate was incubated at 28°C for 72 h, and the cell numbers were counted, the cell viability was calculated.

Cell viability (%) =(A0/A) × 100

A0 represents the number of cell growth in the H_2_O treatment group, the oils group, and the solvent group; while A represents the number of cell growth in the blank control group.

#### Determination of Cell Uptake of Four Vegetable Oils by *S. cerevisiae* Cells

The changes in TPC and TFC were indirectly reflected the uptake of vegetable oils by yeast cells (36). The above mixture without adding H_2_O_2_ was centrifuged at 10,000 g for 5 min; the supernatant was filtered with the membrane (pore size: 0.45 μm), TPC and TFC of the filtrate were tested as C1; after adding H_2_O_2_, the treated method was the same as the method without adding H_2_O_2_, and TPC and TFC of the filtrate were tested as C2. The changes in C1 and C2 were used as the uptake of vegetable oils by yeast cells.

#### Determination of the Protective Effect of Four Vegetable Oils

The protective effect of vegetable oil on cells was measured on a plate containing 0.7% agar YPD medium by cell halo method (37). The cell suspension was diluted 100 times, and added 10 mL into 90 mL of 0.7% agar YPD medium, which was poured into three plates (Φ = 9 cm). After solidification, three filter papers (Φ = 0.5 cm) were placed on the medium's surface, arranged in an equilateral triangle. Adding 2 μL of 50 mg/mL vegetable oil to the top filter paper, and 2 μL of 50 mg/mL vegetable oil and 2 μL of vegetable oil solvent to the right and left filter papers, 1 h later, 2 μL of H_2_O_2_ were added to the right and left filter papers, which was incubated at 28°C for 3 days, and the cell halo was observed. The halo was measured by the cross method, the cell protection rate was calculated.

Cell protection rate (%) = [(B0-0.5)–(B-0.5)]/(B0-0.5) × 100,

Among them, B0 represents the vegetable oil solvent+H_2_O_2_ group, B represents the vegetable oil group+H_2_O_2_, and 0.5 represents the filter paper diameter.

#### Determination of MDA Content, ROS Level, the Enzyme Activity of Four Vegetable Oils

The MDA content of the cells was measured using the MDA kit (BC0020) method. The DCFH-DA kit was used to measure intracellular ROS level changes. A kit (SOD, BC0175; CAT, BC0205; POD, BC0090; GR, BC1160) was used to determine the corresponding enzyme activity.

#### Determination of the Antibacterial Activities of Vegetable Oils

*Staphylococcus aureus, Escherichia coli, Pseudomonas aerugino, Bacillus cereus*, and *Bacillus* subtilis were provided by the college of plant protection, Hainan University. The minimum inhibitory concentration (MIC) of four vegetable oils was measured against five pathogenic bacteria using the multiple dilution method (38).

### Statistics Analysis

Statistical analysis were performed using (SAS 9.1.3; SPSS version 21), Origin pro 9.0 were used for data and image analysis. The mean ± standard deviation (SD) of each repeated experiment. When *p* < 0.05, it is a significant difference.

## Results and Analysis

### GC-MS Analysis of Vegetable Oils

**Figure 1:**
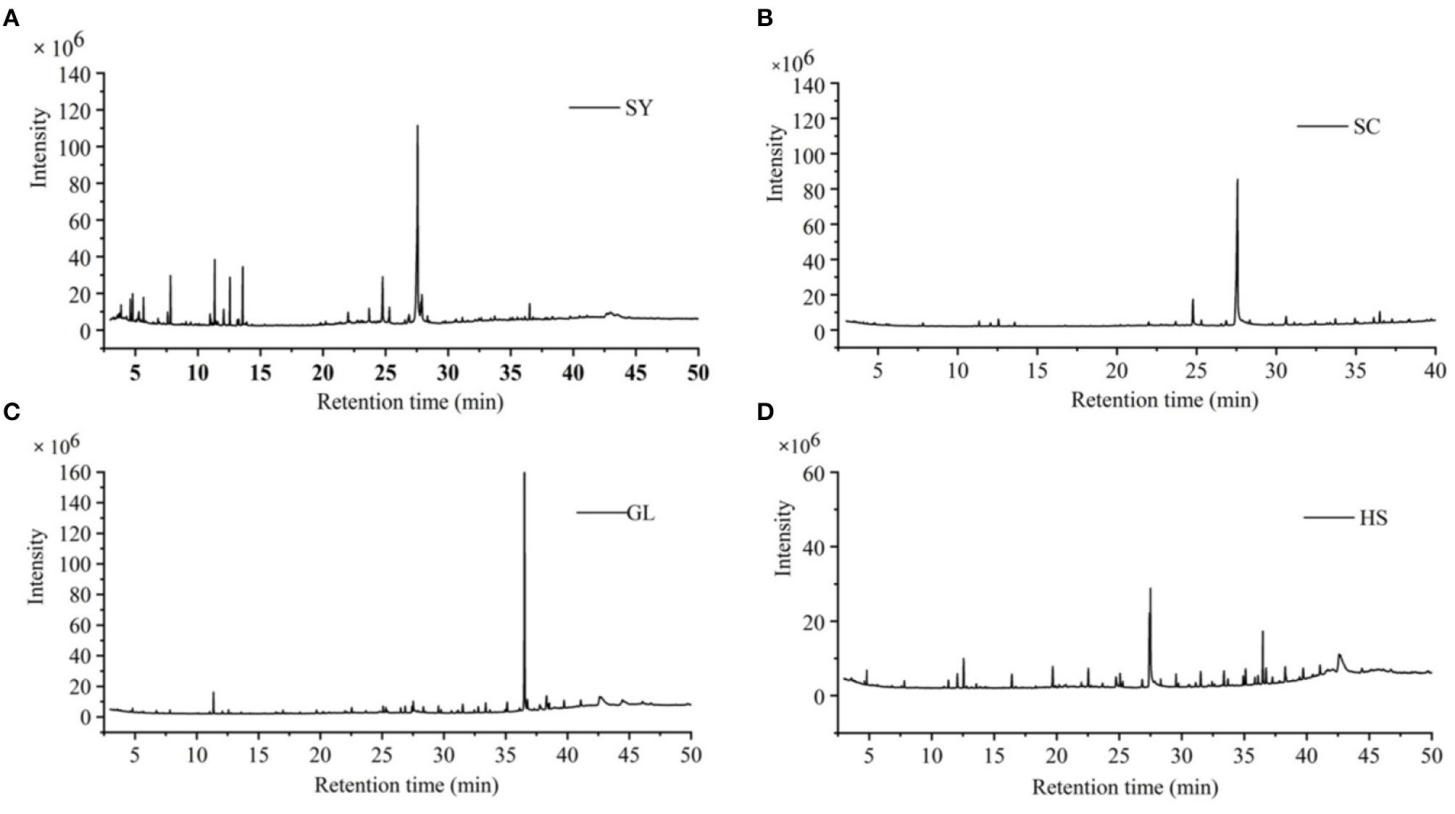
Total ion flow charts of four vegetable oils. **(A)** SY camellia oil from Hainan, **(B)** SC camellia oil from Hainan Guangxi, **(C)** GL olive oil, and **(D)** HS peanut oil.

GC-MS obtained the total ion flow charts of the four vegetable oils (Figure 1). Compared with the GC-MS database, the chemical components of vegetable oils were identified; the peak area normalization method was used to express the compound's content.

Through GC-MS analysis, the major content of SY was Oleic Acid (51.63%), followed by *n*-hexadecanoic Acid (8.41%), methyl oleate (6.59%), a-Amyrin (6.13%), and so on (Table 1); the major content of SC was Oleic Acid (58.02%), followed by *n*-hexadecanoic Acid (6.86%), a-Amyrin (5.29%), Ethyl iso-allocholate (3.38%), etc. (Table 2); the major content of GL was squalene (52.60%), followed by β-sitosterol (9.15%), 2-(3-acetoxy-4,4,14-trimethylandrost-8-en-17-yl)-Propanoic Acid (4.77%), Oleic Acid (4.22%), etc. (Table 3); the major content of HS was Oleic Acid (26.46%), followed by β-sitosterol (14.24%), squalene (6.54%), Oleic acid, eicosyl ester (2.65%), etc. (Table 4).

**Table 1:** Chemical composition analysis of SY vegetable oils.

**No**.	**RT**	**Compounds**	**Formula**	**Molecular mass**	**Area%**
1	21.99	Heptadecane	C_17_H_36_	240	2.50
2	22.76	Non-adecane	C_19_H_40_	268	0.87
3	23.07	2-methyl-octadecane	C_19_H_40_	268	1.02
4	23.69	Octadecane	C_18_H_38_	254	2.63
5	24.40	2,6,10-trimethyl-Tetradecane	C_17_H_36_	240	0.47
6	24.76	*n*-hexadecanoic acid	C_16_H_32_O_2_	256	8.41
7	25.30	Eicosane	C_20_H_42_	282	2.98
8	26.56	(Z)-7-Hexadecenal	C_16_H_30_O	238	0.35
9	26.87	2-methyl-hexadecanol	C_17_H_36_O	256	1.83
10	27.56	Oleic Acid	C_18_H_34_O_2_	282	51.63
11	27.87	methyl oleate	C_19_H_36_O_2_	296	6.59
12	28.34	Octacosane	C_28_H_58_	394	0.83
13	30.63	(Z)-9-octadecamide	C_18_H_35_NO	281	1.00
14	31.14	2,6,10,15-tetramethylheptadecane	C_21_H_44_	296	0.63
15	32.17	dodecyl 2-ethylhexanoate	C_20_H_40_O_2_	312	0.55
16	32.65	2-propenyl decanoic acid	C_13_H_24_O_2_	212	1.22
17	33.73	Tetracosane	C_24_H_50_	338	0.79
18	35.56	(Z, Z)-9,12-octadecadienoic acid	C_18_H_32_O_2_	280	0.70
19	36.13	Heptacosane	C_27_H_56_	380	0.63
20	36.51	Squalene	C_30_H_50_	410	1.94
21	42.98	a-Amyrin	C_30_H_50_O	426	6.13
22	43.53	β-Sitosterol	C_29_H_50_O	414	2.55

**Table 2:** Chemical composition analysis of SC vegetable oils.

**No**.	**RT**	**Compounds**	**Formula**	**Molecular mass**	**Area%**
1	21.99	Heptadecane	C_17_H_36_	240	1.00
2	23.67	Octadecane	C_18_H_38_	254	0.98
3	24.77	*n*-hexadecanoic acid	C_16_H_32_O_2_	256	6.86
4	25.29	Non-adecane	C_19_H_40_	268	1.25
5	26.87	2,6,10-Trimethyl-tetradecane	C_17_H_36_	240	1.43
6	27.54	Oleic Acid	C_18_H_34_O_2_	282	58.02
7	28.34	2-(octadecyloxy)-Ethanol	C_20_H_42_O_2_	314	0.46
8	29.76	10-methyl-non-adecane	C_20_H_42_	282	0.69
9	30.61	Trans-13-octadecenoic acid	C_18_H_34_O_2_	282	2.4
10	31.12	Heneicosane	C_21_H_44_	296	0.55
11	32.45	Octacosane	C_28_H_58_	394	0.95
12	33.71	Pentadecane	C_25_H_52_	352	1.2
13	34.94	Tetracosane	C_24_H_50_	338	1.71
14	36.13	Hentriacontane	C_31_H_64_	436	1.66
15	36.49	Squalene	C_30_H_50_	410	2.39
16	37.28	3-ethyl-5-(2-ethylbutyl)-Octadecane	C_26_H_54_	366	1.64
17	38.36	Heptacosane	C_27_H_56_	380	1.02
18	42.97	a-Amyrin	C_30_H_50_O	426	5.29
19	45.35	Ethyl iso-allocholate	C_26_H_44_O_5_	436	3.38
20	45.79	(±)-1,2-dipalmitin	C_35_H_68_O_5_	568	1.97
21	46.16	3-(octadecyloxy)propyl oleate	C_39_H_76_O_3_	592	1.55

**Table 3:** Chemical composition analysis of GL vegetable oils.

**No**.	**RT**	**Compounds**	**Formula**	**Molecular mass**	**Area%**
1	25.31	Eicosane	C_20_H_42_	282	1.32
2	26.51	Farnesol	C_15_H_26_O	222	0.77
3	26.87	Non-adecane	C_19_H_40_	268	1.07
4	27.52	Oleic Acid	C_18_H_34_O_2_	282	4.22
5	28.34	Heneicosane	C_21_H_44_	296	0.93
6	31.14	2,6,10,15-tetramethyl-Heptadecane	C_21_H_44_	296	0.77
7	32.78	Geranylgeraniol	C_20_H_34_O	290	0.94
8	36.15	Geranyl isovalerate	C_15_H_26_O_2_	238	0.88
9	36.52	Squalene	C_30_H_50_	410	52.60
10	37.78	(all-E)-Oxirane, 2,2-dimethyl-3-(3,7,12,16,20-pentamethyl-3,7,11,15,19-heneicosapentaenyl)	C_30_H_50_O	426	1.82
11	42.65	β-sitosterol	C_29_H_50_O	414	9.15
12	44.45	2-(3-acetoxy-4,4,14-trimethylandrost-8-en-17-yl)-Propanoic acid	C_27_H_42_O_4_	430	4.77
13	46.07	Ethyl iso-allocholate	C_26_H_44_O_5_	436	2.17

**Table 4:** Chemical composition analysis of HS vegetable oils.

**No**.	**RT**	**Compounds**	**Formula**	**Molecular mass**	**Area%**
1	21.97	Octadecane	C_18_H_38_	254	0.81
2	23.67	Heptadecane	C_17_H_36_	240	0.74
3	24.75	*n*-hexadecanoic acid	C_16_H_32_O_2_	256	2.16
4	26.83	Non-adecane	C_19_H_40_	268	1.23
5	27.47	Oleic Acid	C_18_H_34_O_2_	282	26.46
6	28.32	Heneicosane	C_21_H_44_	296	0.60
7	30.58	(Z)-9-octadecamide	C_18_H_35_NO	281	0.61
8	31.12	Octacosane	C_28_H_58_	394	0.62
9	32.14	3-hydroxy-lauric acid	C_12_H_24_O_3_	216	0.44
10	32.43	2,6,10-Trimethyl-tetradecane	C_17_H_36_	240	1.07
11	33.69	Hexacosane	C_26_H_54_	366	1.03
12	35.85	Erucic Acid	C_22_H_42_O_2_	338	1.07
14	36.11	Heptacosane	C_27_H_56_	380	1.24
15	36.49	Squalene	C_30_H_50_	410	6.54
16	37.26	Tritetradecane	C_34_H_70_	478	0.73
17	37.82	2,2,4-Trimethyl-3-(3,8,12,16-tetramethyl-heptanedione-3,7,11,15-tetraphenyl)-cyclohexanol	C_30_H_52_O	428	0.27
18	39.44	cis-(2-Phenyl-1,3-dioxolan-4-yl)methyl 9-octadecenoate	C_28_H_44_O_4_	444	0.47
19	41.68	Campesterol	C_28_H_48_O	400	2.41
20	42.07	(3á,22E)-Ergosta-5,22-dien-3-ol acetate	C_30_H_48_O_2_	440	1.90
21	42.63	β-sitosterol	C_29_H_50_O	414	14.24
22	45.76	Oleic acid, eicosyl ester	C_38_H_74_O_2_	562	2.65
23	46.13	Ethyl iso-allocholate	C_26_H_44_O_5_	436	1.15

### TPC and TFC of Vegetable Oils

With gallic Acid and Rutin as references, the standard curves of TPC and TFC were established, and the fitted linear regression equations were y = 0.0011x + 0.0025, y = 0.0007x + 0.0078, respectively; the correlation coefficients were *R*^2^ = 0.9972, *R*^2^ = 0.9998, which showed that the correlation was excellent. According to the regression equations, the TPC and TFC of four vegetable oils were further calculated (Table 5). Among the vegetable oils, the TFC of SY was the highest (39.50 ± 0.41 mg RE/g DW), followed by GL (35.84 ± 0.08 mg RE/g DW). TPC of SC was the highest (47.05 ± 0.72 mg GAE/g DW), followed by SY (43.8 ± 0.28 mg GAE/g DW); there was no significant difference in TPC between HS and GL.

**Table 5:** The results of TPC and TFC of four vegetable oils.

	**SY**	**SC**	**GL**	**HS**
TPC (mg GAE/g DW)	43.80 ± 0.28^b^	47.05 ± 0.72^a^	38.89 ± 0.71^c^	38.02 ± 0.62^c^
TFC (mg RE/g DW)	39.50 ± 0.41^a^	11.08 ± 0.22^d^	35.84 ± 0.08^b^	19.79 ± 0.36^c^

*The mean ± standard deviation (n = 3). The lowercases were the significant difference at the same column (P < 0.05)*.

### Antioxidant Effect of Vegetable Oil *in vitro*

**Figure 2:**
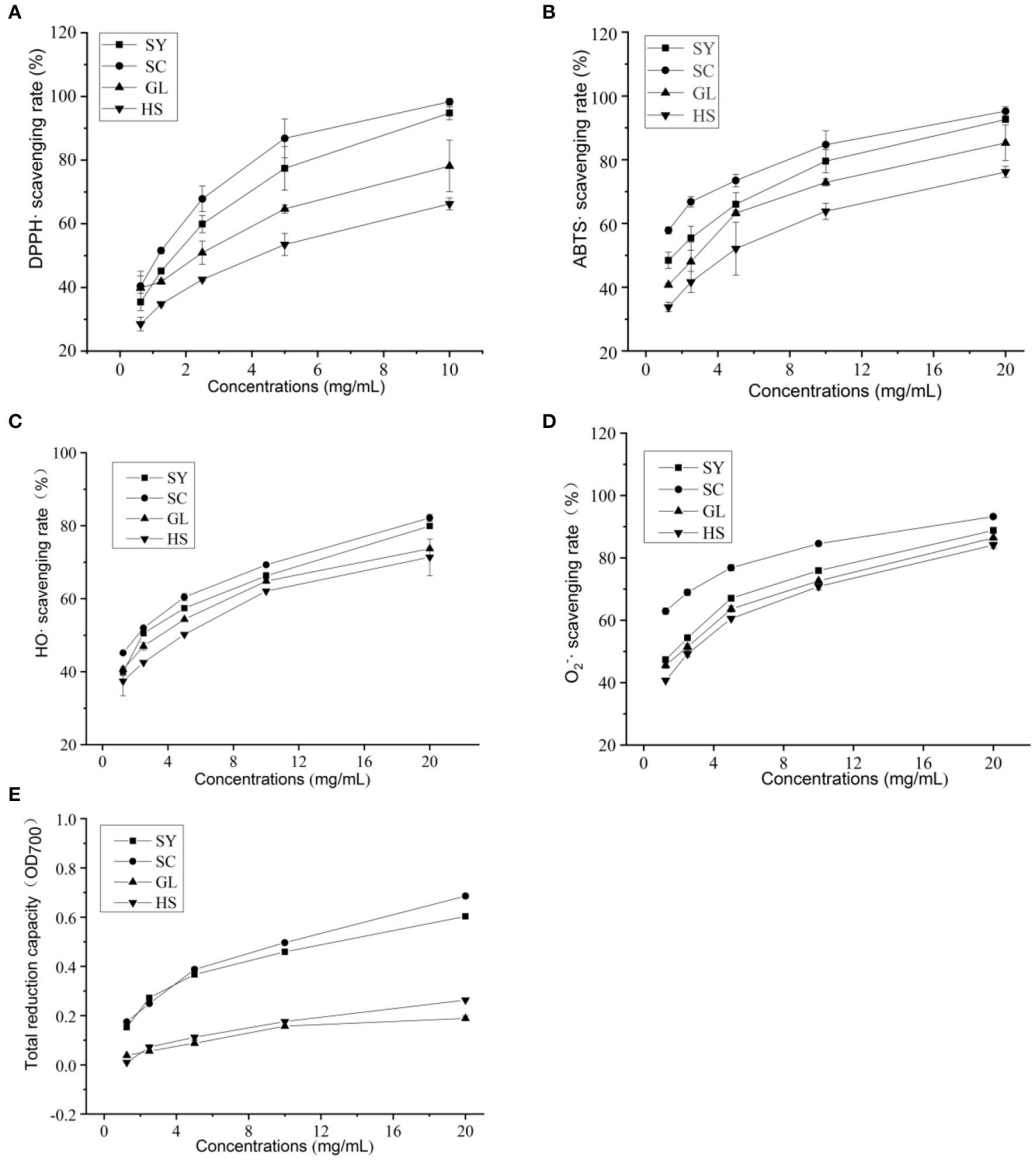
Antioxidant activities of four vegetable oils. **(A)** DPPH·, **(B)** ABTS·, **(C)** HO·, **(D)** ${\text{O}}_{2}^{-}\cdot$, **(E)** total reduction capacity.

The DPPH· scavenging activities were increased with the concentration of vegetable oils increases (Figure 2A). When the concentration was 0.625–2.5 mg/mL, the four vegetable oils' scavenging rate increased rapidly; the concentration was 5–10 mg/mL, the scavenging rate increased slows down. At the same concentration, SC's scavenging rate in the four vegetable oils was the highest; mostly, the scavenging rate was 98.32% at 10 mg/mL. The IC_50_ of each vegetable oil was shown in Table 6. The four vegetable oils' DPPH· scavenging activity was ranked from strong to weak: SC> SY> GL> HS.

**Table 6:** IC_50_ values of four vegetable oils.

**Sample**	**IC**_****50****_ **(mg/mL)**				
	**DPPH·**	**ABTS·**	**HO·**	**${\text{O}}_{2}^{-}\cdot$**	**TRC**
SY	1.37	1.74	2.06	0.62	11.38
SC	1.04	1.00	2.62	1.72	8.85
GL	1.74	2.48	3	1.98	54.82
HS	3.62	4.01	4.01	2.45	57.46
Gallic acid	0.0023	-	-	-	-
BHT	-	0.11	0.04	0.26	0.10

The ABTS· scavenging activities of four vegetable oils were also increased with the increase in vegetable oil concentration (Figure 2B). When the concentration was 1.25–2.5 mg/mL, the four vegetable oils' scavenging rate increased rapidly and then slowly increased. Among them, SC showed the highest scavenging rate. When the concentration was 20 mg/ml, the clearance rate was 95.22%, followed by SY, the scavenging rate was 92.64%. The IC_50_ of each vegetable oil was shown in Table 6. The ABTS· scavenging activity of the four vegetable oils was ranked from strong to weak: SC> SY> GL> HS.

The HO· scavenging activities of four vegetable oils were also increased with the increase in vegetable oil concentration (Figure 2C). When the concentration was 1.25–5 mg/mL, the scavenging rate of the four vegetable oils increased rapidly, and the scavenging rates of SY and SC were similar at 2.5 mg/mL; when the concentration was 20 mg/mL, the scavenging rate of SY was as high as 82.16%, followed by SC, the scavenging rate was 79.92%. The IC_50_ of each vegetable oil was shown in Table 6. The HO· scavenging activity of the four vegetable oils was ranked from strong to weak: SY> SC> GL> HS.

The ${\text{O}}_{2}^{-}\cdot$ scavenging activities of four vegetable oils were also increased with increased vegetable oil concentration (Figure 2D). When the concentration was 1.25–5 mg/mL, the four vegetable oils' scavenging rate increased rapidly. When the concentration was 20 mg/mL, the scavenging rate of SY was the highest (93.22%), followed by SC, GL, and HS The IC_50_ of each vegetable oil was shown in Table 6. The HO· scavenging activity of the four vegetable oils was ranked from strong to weak: SY> SC> GL> HS.

The reduction effect of four vegetable oils on FRAP high-valent iron ions was measured. It was found that the four vegetable oils had significant differences in the reduction effect (Table 7). Among them, SC had the most substantial reducing effect. One mg of SC sample was equivalent to the reducing ability of 197.68 ± 1.54 μM FeSO_4_; followed by SY (138.01 ± 1.99 μM FeSO_4_).

**Table 7:** Comparison of FRAP results of four oil s (20 mg/mL).

**Vegetable oils**	**SY**	**SC**	**GL**	**HS**
Average absorbance	0.37 ± 0.00	0.47 ± 0.00	0.31 ± 0.01	0.25 ± 0.00
μM FeSO_4_/mg DW	138.01 ± 1.99^b^	197.68 ± 1.54^a^	104.93 ± 2.91^c^	70.90 ± 0.87^d^

*The mean ± standard deviation (n = 3). The lowercases were the significant difference at the same column (P < 0.05)*.

The total reducing capacity of vegetable oils increased with vegetable oil concentration (Figure 2E). In comparison, the total reducing capacity of SY and SC vegetable oils were relatively close, and the total reducing capacity of GL and HS vegetable oils were relatively close. Overall, SC showed the strongest total reducing capacity. The IC_50_ of each vegetable oil was shown in Table 6. The total reducing ability of the four vegetable oils is SC> SY> GL> HS.

### Protective Effects of Vegetable Oils on Yeast Cells Under H_2_O_2_ Stress

#### The Protective Effect of Vegetable Oils on the Yeast Cells

**Figure 3:**
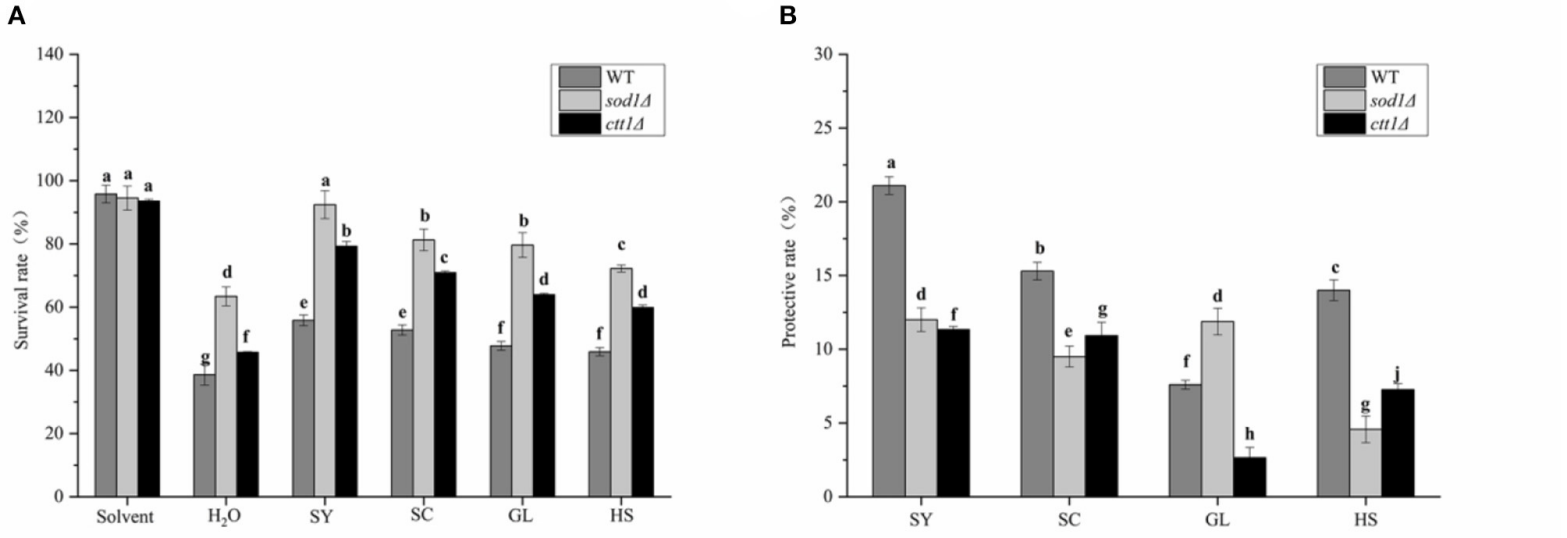
The protective effect of vegetable oils on yeast cells. **(A)** the survival rate of yeast cells; **(B)** the protective effect on yeast cells; Lower case letters have significant differences at *p* < 0.05.

Wild-type (WT) and genetic mutant (*sod1*Δ, *cttl*Δ) yeast cells were used to test cell viability. The oxidative stress of yeast cells stressed by H_2_O_2_ was alleviated by adding SY, SC, GL, and HS (Figure 3A). The survival rate of yeast cells was the lowest in the H_2_O treatment group, and was the highest in the solvent group without H_2_O_2_ stress, which showed that the solvent had little effect on the yeast cells. SY, SC, GL, and HS could better alleviate the oxidative damage caused by H_2_O_2_ in WT, *sod1*Δ, and *ctt1*Δ cells. In particular, the survival rate of SY was the highest in yeast cells under H_2_O_2_ stress, the increased value of the survival rate, in turn, was 17.19, 28.98, 33.54%, respectively. Also, the survival rates of *sod1*Δ and *ctt1*Δ cells were significantly higher than the other vegetable oils. Besides, SC also showed a better effect on the cell's survival under stress. In comparison, the impact on *sod1*Δ and *ctt1*Δ cells was higher than that of WT cells. Compared with the control, vegetable oils' protective effect on yeast cells was found treated with H_2_O_2_ (Figure 3B). SY showed the most obvious protective effect. The protective rates for WT, *sod1*Δ, and *ctt1*Δ were 21.07, 12.01, and 11.34%, respectively. Secondly, the protective rates of SC were also more obvious. It was 15.27, 9.51, 10.93%, respectively. Comparison between different yeast cells, the vegetable oils showed an excellent protective effect on WT cells.

#### Yeast Cells' Uptake to Total Phenols and Total Flavonoids of Vegetable Oils

**Figure 4:**
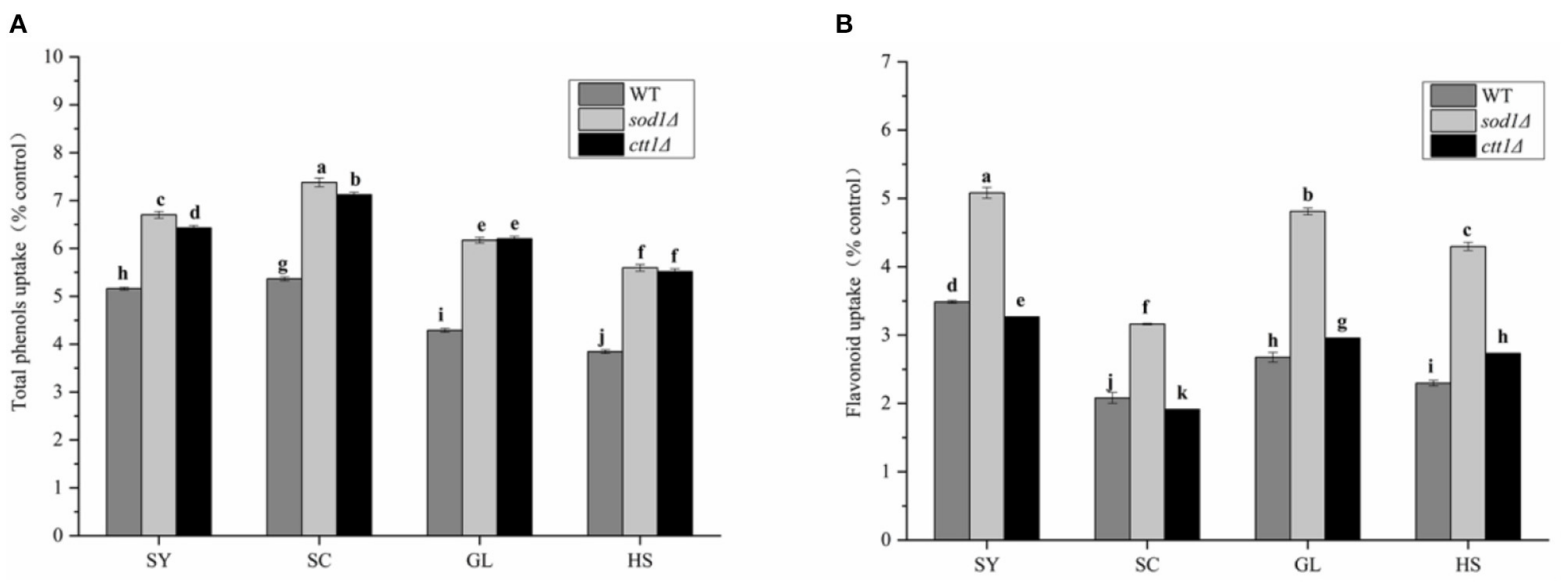
Total phenols and Flavonoid intake of yeast oil cells against four oils. **(A)** Total phenols **(B)** total Flavonoids; Lower case letters indicate the difference in entire phenolic content at *p* < 0.05.

WT, *sod1*Δ, and *ctt1*Δ yeast cells showed the highest uptake to total phenols of SC (Figure 4A), and the uptake content was 5.37 times, 7.38 times, and 7.12 times that of the blank, respectively; followed by SY, the uptake content was 5.16 times, 6.70 times and 6.43 times that of the blank. Compared with three yeast cells, the uptake to total phenols by *sod1*Δ cell was the highest, followed by *ctt1*Δ cell, and WT cell.

Also, WT, *sod1*Δ, and *ctt1*Δ yeast cells showed the highest uptake to total flavonoids of SY (Figure 4B), and the uptake content was 3.48 times, 5.08 times, and 3.27 times that of the blank, respectively; The three kinds of yeast cells had the lowest intake to total flavonoids of SC The same as total phenols, *sod1*Δ cells showed the highest uptake of total flavonoids. These results were the same as the TPC and TFC of the oils.

#### Effect of Vegetable Oils on MDA Content of Yeast Cells

**Figure 5:**
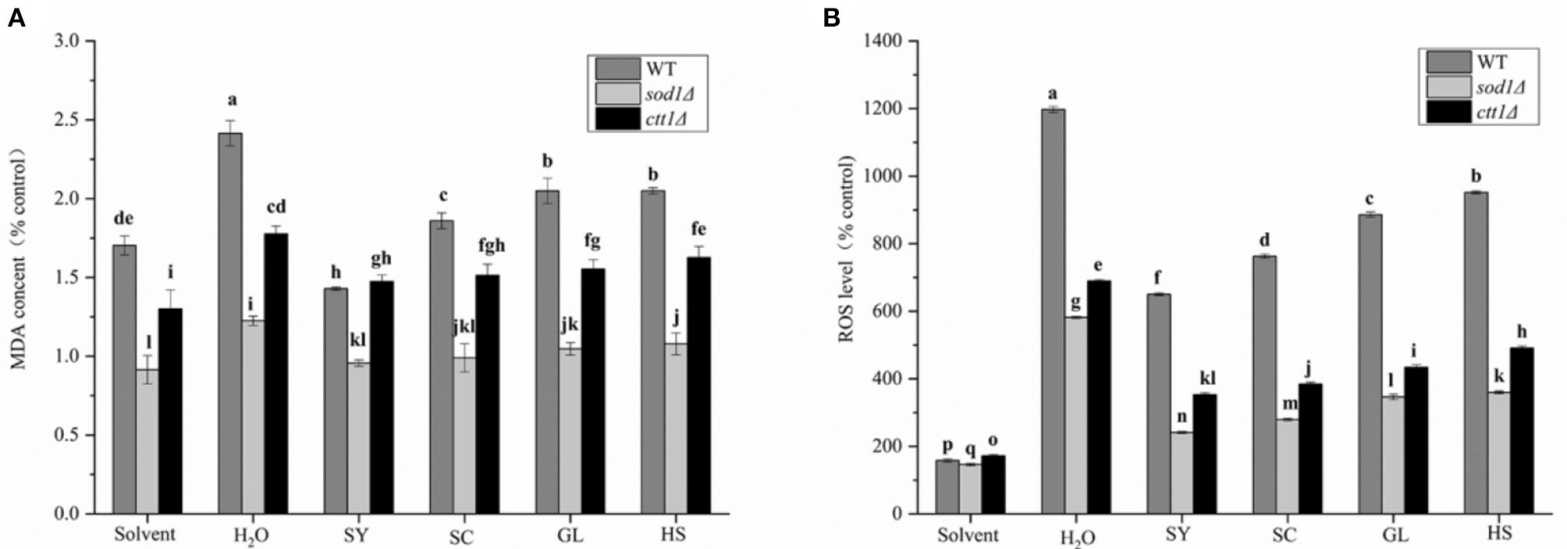
MDA content and ROS level of four oils in yeast cells. **(A)** MDA content, **(B)** ROS level. Lower case letters indicates the difference at *p* < 0.05.

Under oxidative stress, the MDA content of the yeast cells will increase. So, treated yeast cells with H_2_O_2_, the MDA content of the cells increased dramatically in the H_2_O treatment group (Figure 5A), the MDA content of WT, *sod1*Δ, and *ctt1*Δ cells were 2.42, 1.23, and 1.78 times that of the cells without H_2_O_2_ treatment; the changes in MDA content under vegetable oils protection, the H_2_O treatment group showed a significant difference, especially, the SY was significantly different from the other three vegetable oils. The MDA content was decreased from 2.42 times to 1.43 times, and the MDA content of sod1Δ cells was reduced from 1.23 times to 0.96 times. The MDA content of *ctt1*Δ cells decreased from 1.78 times to 1.48 times. Compared with the effect of vegetable oils on the three yeasts, the MDA content in WT cells was the most obvious, especially, under SY and SC protection, the MDA content was decreased to 59.0% and 83.1% compared with the H_2_O treatment group.

#### Effect of Vegetable Oil on the ROS Level of Yeast Cells

When cells are subjected to external stimulation to produce oxidative stress, the intracellular ROS level increase significantly. The intracellular ROS level was significantly increased under H_2_O_2_ treatment (H_2_O treatment group), the ROS levels of WT, *sod1*Δ, *ctt1*Δ cells were 11.98 times, 5.82 times, 6.90 times compared with the blank, respectively (Figure 5B). Under the protection of the vegetable oils, the changes in ROS level in the cells were reduced. Mostly, the protective effect of SY was the most obvious. The ROS level of WT cells was 6.51 times compared with the blank, which was about 54.3% of the H_2_O treatment group; the ROS level of *sod1*Δ cells was 2.41 times of the blank, which was approximately 41.4% of the H_2_O treatment group; the ROS level of *ctt1*Δ cells was 3.53 times of the blank, which was about 51.1% of the H_2_O treatment group. Secondly, the protective effect of SC showed an excellent effect. Comparison with different yeast cells, the protective effect of the oils to *sod1*Δ cells was the best, followed by *ctt1*Δ cells.

#### Effect of Vegetable Oil on Enzyme Activity in Yeast Cells

**Figure 6:**
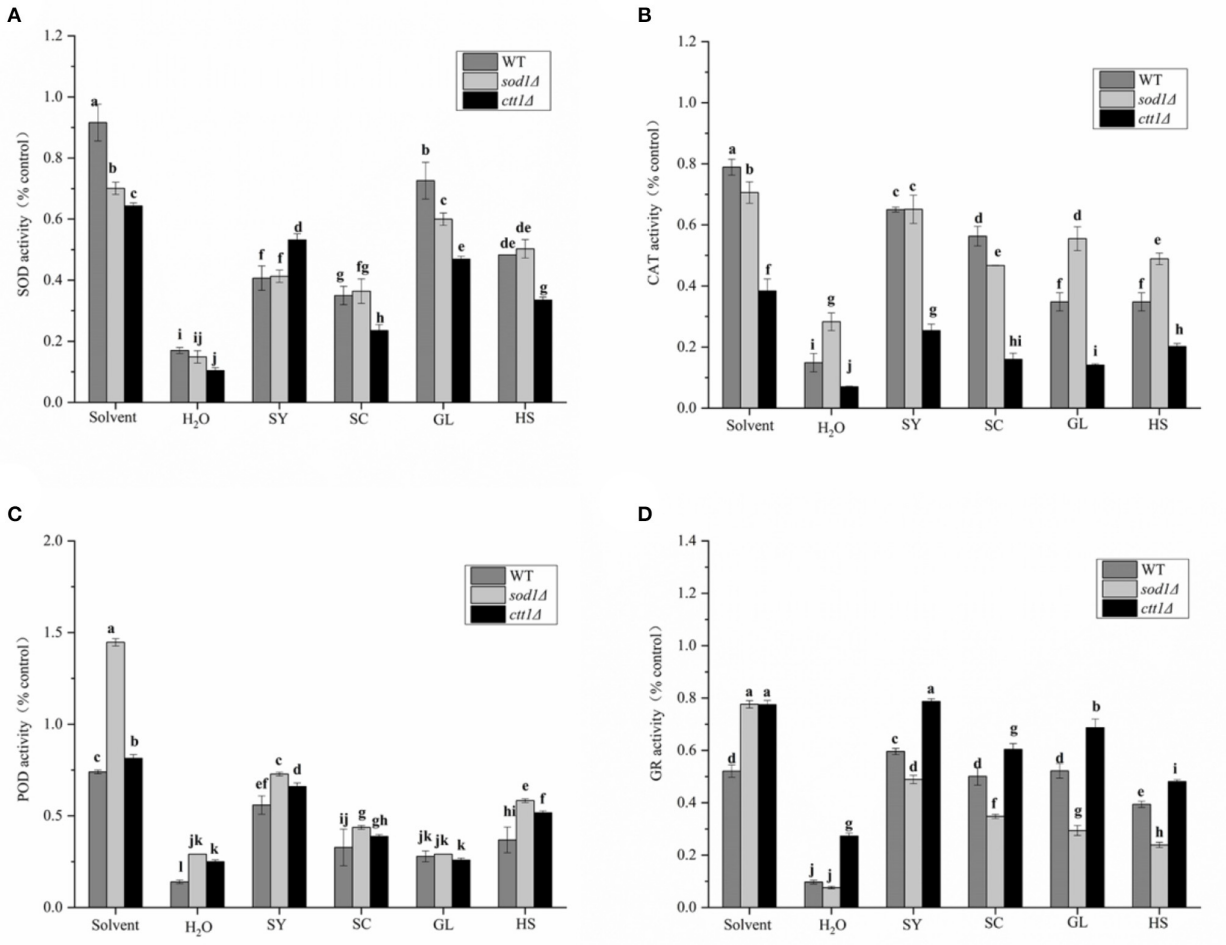
Effects of the oils on enzyme activity of yeast cells. **(A)** SOD, **(B)** CAT, **(C)** POD, **(D)** GR the results of enzyme activity are expressed as the ratio of cells treated with oil, H_2_O_2_ treated cells, and non-H_2_O_2_ treated cells. Lower case letters indicate the difference at *p* < 0.05.

It was found that the H_2_O_2_ treatment (H_2_O treatment group) could cause the intracellular SOD enzyme activity to be significantly lower than the solvent group (Figure 6A). The SOD enzyme activity of WT, *sod1*Δ, *ctt1*Δ cells was 16.99, 14.91, 10.38% of the control, respectively. Under the protection of the vegetable oils, the SOD enzyme activity was all increased. The protective effect of GL was excellent, SOD enzyme activity of WT, *sod1*Δ, *ctt1*Δ cells was 72.63, 60.00, and 46.87% of the control, respectively. Followed by HS, SOD enzyme activity of WT, *sod1*Δ, *ctt1*Δ cells was 40.68, 42.26, and 33.46% of the control, respectively. The protective effect of SY to ctt1Δ cells was the better than that of WT, *sod1*Δ cells.

The CAT enzyme activity of WT, *sod1*Δ, *ctt1*Δ cells was 14.93%, 28.33% while 7.05% of the control group (Figure 6B). Under the protection of the vegetable oils, the CAT enzyme activity of the yeast cells was increased. The CAT enzyme activity of SY in WT, sod1Δ, and ctt1Δ cells was 64.97, 65.14, and 25.39% of the control, respectively. Following SC treatment, the CAT enzyme activity of WT, sod1Δ, and ctt1Δ cells was 56.34, 46.74, and 16.00% of the control group. Different yeast cells, For example, SY, GL, and HS showed the most significant effect on *sod1*Δ cells, while SC showed the most significant effect on WT cells.

The POD activity of WT, *sod1*Δ, *ctt1*Δ cells was 13.97%, 29.13% while 24.99% of the control (Figure 6C). When the vegetable oils were added, the POD enzyme activity of the yeast cells was increased. Among them, the protective effect of SY was the most obvious. The POD enzyme activity in WT, *sod1*Δ, and *ctt1*Δ cells was 55.89, 72.83, and 65.97% of the control. After HS treatment, the POD enzyme activity of WT, *sod1*Δ, and *ctt1*Δ cells was 36.94, 58.26, and 51.75% of the control.

The GR enzyme activity of WT, *sod1*Δ, *ctt1*Δ cells was 9.65, 7.64, 27.27% of the control (Figure 6D). When the vegetable oils were added, the three yeast cells' GR enzyme activity was all increased. Among them, the protective effect of SY treatment was the most obvious. The GR enzyme activity in WT, *sod1*Δ, and *ctt1*Δ cells was 59.63, 48.90, and 78.75% of the control. The vegetable oils had the same order (*ctt1*Δ, WT, *sod1*Δ) to affect the GR enzyme activity.

#### Inhibitory Effect of the Vegetable Oils on Five Bacteria

At the tested concentration, SY had no inhibitory effect on the five bacteria (Table 8). SC had inhibitory effects on *E. coli* and *B. cereus*, and the MIC of both was 20 mg/mL. GL had an excellent inhibitory effect on five bacteria, the MIC was the same 5 mg/mL against *E. coli* and *B. cereus*; the MIC was 10 mg/mL against *S. aureus*, and the MIC was 20 mg/mL against *B. subtilis*. HS had an excellent inhibitory effect on *B. cereus*, and the MIC was 10 mg/mL. But, treated with Streptomycin (5 mg/mL), five bacteria did not grow. Thus, the inhibitory effect of the oils was not good.

**Table 8:** Inhibition of five pathogenic bacteria by four oils.

**Concentration (mg/mL)**		**Test strain**					**Streptomycin mass concentration (mg/mL)**
		***E. coli***	***P. aeruginosa***	***B. cereus***	***S. aureus***	***B. subtilis***	
SY	40	++	++	++	++	+	
	20	++	++	++	++	++	
	10	++	++	++	++	++	
	5	++	++	++	++	++	–
SC	40	–	+	–	+	+	
	20	–	++	–	++	++	
	10	+	++	++	++	++	
	5	++	++	++	++	++	–
GL	40	–	–	–	–	–	
	20	–	+	–	–	–	
	10	–	++	–	–	+	
	5	–	++	–	+	+	–
HS	40	–	+	–	+	–	
	20	–	++	–	++	+	
	10	+	++	–	++	++	
	5	++	++	+	++	++	–

*“–” aseptic growth; “+” a small number of colonies grow; “+ +” many colonies grow*.

## Discussion

According to the GC-MS analysis results, the chemical composition of the vegetable oils was not the same. Oleic Acid, Squalene, and Alkane (19, 20-carbon chain) were detected in four vegetable oils. Oleic Acid was the major in SY, SC, and HS, but the content of oleic Acid was lower than that of Feás's and Zhou's reports (12, 39); β-sitosterol was detected in SY, GL, and HS The major content of GL was squalene, and squalene showed important bioactivities (40). This study showed that GL had the best antibacterial effect against five bacteria; also, HS's antibacterial effect was good. Maybe, the content of squalene was higher than other oils. Squalene was easily oxidized, so GL's protective effect was less than that of the other oils. And SC had inhibitory effects, MIC *for B. cereus* was 20 mg/mL, which was better than Feás's report, but the MIC of SC for *E. coli* was not good as Feás's report (12). There are differences in tea seeds from different sources, regions, and climatic conditions, as well as in the vegetable oils obtained by different pressing methods. Thus, this is a preliminary result because the four vegetable oils were selected at random and not sampled in large numbers.

TPC of SC and TFC of SY were the highest in the four vegetable oils. This result was consistent with the reference (41), and TPC of SC and SY was higher than that reported by Wang et al. (2.18 mg GAE/g oil) (13). It was possible that the tea seeds of our sample were physically pressed by shelling, while Wang's sample was extracted with supercritical carbon dioxide. The antioxidant capacity was also outstanding. Among them, SC exhibited the strongest scavenging ability for DPPH·, ABTS·, FRAP, and TRC. At the same time, the scavenging ability for HO· and ${\text{O}}_{2}^{-}\cdot$ was also prominent; then, SY showed the excellent scavenging ability for DPPH·, ABTS·, FRAP, and TRC. It was pointed out that free radical scavenging ability was often positively correlated with TPC (42). This study supported this view. And DPPH· scavenging activity of SC and SY was higher that reported by Wang et al. (10), which was due to the higher TPC of SC and SY. At the same time, this study's results proved that the free radical scavenging ability was positively correlated with the TFC of vegetable oils. To a certain extent, excessive H_2_O_2_ can induce oxidative stress and lead to membrane damage in cells (43). MDA content changes can indirectly prove membrane damage in the cell (44, 45). Treated yeast cells (wild-type and mutant) with H_2_O_2_, the MDA content and ROS level of SY, SC were significantly reduced, which was alleviated the oxidative damage induced by H_2_O_2_ and increased the survival rate of yeast cells. This conclusion was consistent with Li's reports, and verified the guess “the oil of tea seed may act as a prophylactic agent to prevent free radical related diseases” (11). Also, the tested yeast cells showed the highest intake of total phenols of SC and the highest intake of total flavonoids of SY, which was the same as the corresponding TPC and TFC of the these vegetable oils. We speculated that the content of antioxidants in vegetable oils could affect the uptake of yeast cells. At the same time, SY showed the best protective effect on yeast cells, and it was the most significant about the reduction of intracellular ROS levels and MDA content. *Saccharomyces cerevisiae* is a good model organism in antioxidant research. In this paper, for the first time, yeast was used to clarify the antioxidant effect of camellia oil, which was consistent with the results of antioxidant activity *in vitro*. Phenolic acids and flavonoids had strong antioxidant activity, and TPC and TFC of SC, SY were significantly higher than those of other oils, so, SC, SY showed excellent antioxidant activities *in vitro* and *in vivo* in this study.

On the whole, many indexes of SY in the antioxidant determination were significantly lower than those of SC Exogenous antioxidants can also alleviate the damage of intracellular peroxides; the cell's antioxidant system also plays a vital role, mainly including antioxidant enzymes and small molecules antioxidant substances. So, the enzyme activities of CAT, POD, GR treated with SY were higher than that of other oils.

## Conclusion

By comparison, many components were the same as in SY and SC, such as oleic Acid, *n*-hexadecanoic Acid, a-Amyrin, only the content was not consistent; those were different from GL. Also, TPC and TFC of SY and SC were higher than that of the other oils. *In vitro*, SY showed the strongest HO· and ${\text{O}}_{2}^{-}\cdot$ scavenging activity, and SC exhibited excellent DPPH· and ABTS· scavenging activity and the reducing abilities. *In vivo*, SY showed excellent protective effect on *S cerevisiae* cells, decreased MDA content and ROS level, inhibited CAT, POD and GR enzyme activity, followed by SC. The antibacterial activity showed GL had a broad-spectrum inhibited activity. Thus, the results provided a reference for the selection of edible vegetable oils in the future.

## Data Availability Statement

The raw data supporting the conclusions of this article will be made available by the authors, without undue reservation.

## Author Contributions

LW and YL conceived and designed the experiments. LW, SA, and YL wrote the manuscript. All authors were contributed the experiments and analyzed the data.

## Conflict of Interest

The authors declare that the research was conducted in the absence of any commercial or financial relationships that could be construed as a potential conflict of interest.
